# Dopamine receptor repertoire of human granulosa cells

**DOI:** 10.1186/1477-7827-5-40

**Published:** 2007-10-25

**Authors:** Veronica Rey-Ares, Nikolai Lazarov, Dieter Berg, Ulrike Berg, Lars Kunz, Artur Mayerhofer

**Affiliations:** 1 Anatomisches Institut, Ludwig-Maximilians-Universität (LMU), München, Germany; 2 Assisted Reproductive Technologies Bogenhausen, München, Germany

## Abstract

**Background:**

High levels of dopamine (DA) were described in human ovary and recently evidence for DA receptors in granulosa and luteal cells has been provided, as well. However, neither the full repertoire of ovarian receptors for DA, nor their specific role, is established. Human granulosa cells (GCs) derived from women undergoing in vitro fertilization (IVF) are an adequate model for endocrine cells of the follicle and the corpus luteum and were therefore employed in an attempt to decipher their DA receptor repertoire and functionality.

**Methods:**

Cells were obtained from patients undergoing IVF and examined using cDNA-array, RT-PCR, Western blotting and immunocytochemistry. In addition, calcium measurements (with FLUO-4) were employed. Expression of two DA receptors was also examined by in-situ hybridization in rat ovary. Effects of DA on cell viability and cell volume were studied by using an ATP assay and an electronic cell counter system.

**Results:**

We found members of the two DA receptor families (D_1_- and D_2_ -like) associated with different signaling pathways in human GCs, namely D_1_ (as expected) and D_5_ (both are Gs coupled and linked to cAMP increase) and D_2_, D_4_ (Gi/Gq coupled and linked to IP3/DAG). D_3_ was not found. The presence of the trophic hormone hCG (10 IU/ml) in the culture medium for several days did not alter mRNA (semiquantitative RT-PCR) or protein levels (immunocytochemistry/Western blotting) of D_1,2,4,5_ DA receptors. Expression of prototype receptors for the two families, D_1_ and D_2_, was furthermore shown in rat granulosa and luteal cells by in situ hybridization. Among the DA receptors found in human GCs, D_2_ expression was marked both at mRNA and protein levels and it was therefore further studied. Results of additional RT-PCR and Western blots showed two splice variants (D_2_L, D_2_S). Irrespective of these variants, D_2_ proved to be functional, as DA raised intracellular calcium levels. This calcium mobilizing effect of DA was observed in the absence of extracellular calcium and was abolished by a D_2_ blocker (L-741,626). DA treatment (48 h) of human GCs resulted in slightly, but significantly enlarged, viable cells.

**Conclusion:**

A previous study showed D_2_ in human GCs, which are linked to cAMP, and the present study reveals the full spectrum of DA receptors present in these endocrine cells, which also includes D_2_-like receptors, linked to calcium. Ovarian DA can act thus via D_1,2,4,5_, which are co-expressed by endocrine cells of the follicle and the corpus luteum and are linked to different signaling pathways. This suggests a complex role of DA in the regulation of ovarian processes.

## Background

High levels of the catecholamine dopamine (DA) were described in human ovary [1,2], raising questions about its role in the female gonad. Several possibilities have so far been suggested. It has been reported that monkey (*Macaca mulatta*) oocytes are able to take up DA and use it as precursor for the synthesis of noradrenaline (NE) [3], with the help of dopamine-β-hydroxylase (DBH), expressed by oocytes. NE may then be able to interact with ovarian alpha- and beta-receptors present on granulosa cells (GCs) of the follicle [2,4,5]. Furthermore, bovine luteal cells are reported to perform synthesis of NE in a similar way [6] and presumably this occurs also in intraovarian nerve fibers and neuron-like cells [7-9], which likewise express DBH. Thus, ovarian DA can be viewed as a precursor for NE in oocytes, endocrine cells and nerve cells and may have a plethora of functions (for example, steroid production and induction of follicle-stimulating hormone receptors) [9-11].

Nevertheless, recent publications have hinted to a yet different function. DA can, in general, act via its five different receptor subtypes (D_1_-D_5_), which are organized in two families (D_1_-like/D_2_-like) and one of them, D_1_, has been described in human luteinizing GCs [12,13] and more recently in endocrine cells of horse ovary [14]. In human GCs, isolated from preovulatory follicles of women undergoing *in vitro *fertilization (IVF) procedures, activation of D_1 _by selective agonists, as well as treatment with DA itself, did not affect progesterone synthesis [12]. D_1 _activation was rather associated with cAMP elevations and increased phosphorylation of DARPP-32 (DA and cAMP regulated phospho-protein of MR 32,000), which is a down-stream third messenger of DA, but also with other signaling molecules in ovary and other tissues [12,13,15-18].

Whether other receptors for DA are present in ovary and on GCs and may thus provide a basis for its action, has as yet not been thoroughly examined. However a recent description of D_2 _in horse granulosa and luteal cells [14] prompted us to examine whether this and other DA receptors are also present in rat and human ovary. For functional studies we focused on human GCs.

## Methods

### Human cells: GC preparation and culture

Human GCs were obtained from follicular aspirates of women undergoing IVF, as described [12,13,19-21]. They were separated by centrifugation at 560 × g for 3 min and subsequent washing in serum-free DMEM/Ham's F-12 medium (1:1, Sigma, Deisenhofen, Germany). Washed cells were resuspended in culture medium supplemented with penicillin (100 U/ml), streptomycin (100 μg/ml), and 10% fetal calf serum as previously described [20]. The use of the cells for scientific experiments had been approved by the ethic's committee of the University of Munich (LMU), and written consent of the patients was obtained. For Ca^2+ ^imaging and immunocytochemistry experiments, cells were cultured on glass cover slips, otherwise in plastic dishes or well plates in a humidified atmosphere with 5% CO_2 _at 37°C. Cells were used for the experiments described either on day 3 or day 4 after isolation. One group of cells was cultured with basal medium, which for the other group was supplemented with hCG (Sigma, 10 IU/ml; from the 1^st ^day of culture).

### cDNA arrays

Cells cultivated for 3 days were harvested and total RNA was prepared as previously described [9,13,19,20] using RNEasy kit (Qiagen, Hilden, Germany). A total of 5 ng was used for the GEArray Q Series Human Neuroscience-1 Ion Channel & Transporter Gene Array (SuperArray Bioscience Corporation, Biomol, Hamburg Germany), as described [20], following the instructions of the manufacturer. The array included D_1_, D_2_, D_3_, D_5_, but not D_4_, in quatriplicate spots. Negative controls consisted of puc18 plasmid cDNA. Chemiluminescence signals were recorded.

### RT-PCR

Total RNA from several batches of cultured GCs (3 and 4 days after isolation) was prepared using RNEasy kit (Qiagen). As previously described [12,22], total RNA (200–500 ng) was subjected to reverse transcription, using random primers (pdN6) and Superscript-RT II (Life Technologies, Karlsruhe, Germany). Commercial human brain cDNA (BD CLONTECH, Inc., Heidelberg, Germany) was used in addition.

Informations about the oligonucleotid primers for DA receptors used are summarized in Table 1. The primers for cyclophilin were identical to the ones described in [22]. As in human, two isoforms of the D_2 _are generated by alternative splicing of exon 6 of the pre-messenger RNA, changing the length of the third cytoplasmic loop involved in the coupling to G proteins [23,24]. In order to be able to distinguish between these variants, we used a semi-nested, second PCR amplification approach. To be able to detect the long form, we employed a further antisense oligonucleotide primer complementary to the cDNA sequence of the 6^th ^exon present only in the long form (see Table 1: nested D_2 _long: 5'- ACT GGG AAA CTC CCA TT-3'). We used the antisense primer (see Table 1: nested D_2 _common: 5'-GAG CAT CTC CAT CTC CA-3') complementary to a common region of both receptor splice variants. With this approach a 261 bp product would correspond to the D_2L _and a 173 bp product to the D_2S_.

**Table 1:** Informations about oligonucleotide primers used for PCR amplification: Sense, antisense and antisense nested primer sequences, as well as their positions in the published D_1_-D_5 _sequences are provided.

**Human dopamine receptor D_1_**	**Sequence**	**Genebank accession number/location of primer**
		NM_000794
sense	5'-CTG AAG ACT CTG TCG GTG A-3'	(1756–1774)
antisense	5'-ACT CAC CGT CTC TAT GGC A-3'	(2031-2013)
nested	5'TGT AGC ATC CTA AGA GGG T-3'	(1990-1972)

**Human dopamine receptor D_2_**		NM_000795
sense	5'-TTC TAC GTG CCC TTC AT-3'	(757–772)
antisense	5'-GGT CTG GAT CTC AAA GA-3'	(1200-1184)
nested D_2 _common	5'-GAG CAT CTC CAT CTC CA-3'	(1017-1000)
nested D_2 _long	5'- ACT GGG AAA CTC CCA TT-3'	(959-943)

**Human dopamine receptor D_3_**		NM_000796
sense	5'-GGT ACT GGC CTT TGC TGT GTC C-3'	905–926
antisense	5'-ATC CTT TTC CGT CTC CTT TGT TTC-3'	1099-1076
nested	5'-CCA AAG GGC AGG TAG AAG GAC-3'	1036-1016

**Human dopamine receptor D_4_**		NM_000797
sense	5'-CCT TGC GGC TCC AAC TGT G-3'	913–931
antisense	5'-AGC GCC TGC GTG ATG TGC-3'	1112-1095
nested	5'-GAA GGC CCC GAC CAC CAC-3'	1065-1048

**Human dopamine receptor D_5_**		NM_000798
sense	5'-GGG CAG TTC GCT CTA TAC CAG-3'	88–108
antisense	5'-CAG GAA AAG GTC TGA CAC GG-3'	321-302
nested	5'-CAG AGA CAC GAT GAA GAC GTT-3'	300-280

In order to be able to evaluate at least semiquantitatively possible changes after treatment of the cultures with hCG, or to take into account small loading differences, in all samples cyclophilin was co-amplified, as described before [22].

The PCR reaction products were separated on 2% agarose gels and visualized with ethidium bromide. The identities of all PCR products were verified by direct sequencing using one of the specific primers [20].

### Measurements of intracellular Ca^2+ ^concentrations

Ca^2+ ^measurements were performed on human GCs (day 3 or 4) cultured on glass cover slips, as described [21,25]. Two groups of cells were examined, namely cells kept without or treated with hCG (10 IU/ml; since the 1^st ^day of culture). Briefly, the medium was replaced by fresh serum-free DMEM/Ham's F-12 medium containing 5 μM fluo-4 AM (Molecular Probes, Eugene, OR, USA) and cells were loaded for 30 min at 37°C and 5% CO_2_. Finally, cells were washed in extracellular solution (140 mM NaCl, 3 mM KCl, 1 mM CaCl_2_, 1 mM MgCl_2_, 10 mM HEPES and 10 mM glucose; pH 7.4) and were put into a recording chamber mounted on a TCS SP2 confocal microscope (Leica Microsystems, Bensheim, Germany). DA and carbachol (Sigma) were applied in 0.1 μM and 100 μM, respectively. The D_2 _selective antagonist used was L-741,626 (1 nM, Tocris Cookson Inc. Missouri, USA). Changes in fluorescence intensity were monitored for 10 min by sampling every 2 s at excitation and emission wavelengths of 488 nm and 520 ± 20 nm, respectively. The Ca^2+ ^free experiments were done with extracellular solution without Ca^2+ ^plus EGTA (2 mM).

### Immunocytochemistry

The cellular distribution of D_1,2,4,5 _proteins in cultured GCs was determined by immunocytochemistry using commercially available polyclonal antisera (rabbit anti-D_1 _receptor, R&D, Berkeley, CA, AS-3512G, 1:500; rabbit anti-D_2_, BioTrend Chemikalien Gmbh, Köln, Germany, AS-3526S, 1:500; rabbit anti-D_4_; Chemicon International Inc., Temecula, CA, AB9422, 1:200; rabbit anti-D_5_, R&D, Berkeley, CA, AS 3552G, 1:200). GCs were fixed on slides (Zamboni's fixative) and used directly after rinsing in 10 mM PBS (pH 7.4). Incubation with the antiserum was carried out overnight in a humidified chamber. A fluorescein isothiocyanate-labeled secondary goat anti-rabbit antiserum was used. For control purposes, the first antiserum was omitted, and incubations with normal rabbit serum were carried out instead. Sections were examined with a Zeiss Axiovert microscope (Zeiss, Oberkochen, Germany), equipped with a fluorescein filter set.

### Western blot analyses

Western blotting was performed as previously described [12,13,18]. In brief, GCs cultured with or without hCG (10 IU/ml for up to 4 days) were harvested, frozen, thawed, homogenized in 62.5 mM Tris-HCl buffer (pH 6.8) containing 10% sucrose and 2% SDS, sonicated, and heated (95°C for 5 min) in the presence of 10% mercaptoethanol. Samples (15 μg/lane) were separated electrophoretically on 10% or 12.5% SDS-polyacrylamide gels (SDS/PAGE). Proteins were transferred onto nitrocellulose membranes and probed with D_1 _and D_2 _antisera, as used for immunocytochemistry (1:1,000 dilution). Immunoreactivity was detected using peroxidase-labeled antisera (1:3,000, Dianova, Hamburg, Germany) and enhanced chemiluminescence, as described [12,13,18] (Amersham Buchler, Braunschweig, Germany). In some cases, the blots were digitized, and integrated optical densities of the bands were determined using an edited version of the program NIH Image, as described previously [12,13,18]. To be able to detect small differences in loading, blots were also probed with mouse anti beta-actin antibody (Sigma). Three independent experiments were evaluated.

### Cell volume and viability

To determine whether DA can affect cell viability or volume, cells were seeded in duplicate in 24-well tissue culture plates and stimulated for 48 h with or without DA (10 μM). After that the cells were trypsinized and the average cell volume was examined using an electronic Coulter counter (CASY-TT, Schärfe System, Reutlingen, Germany).

To determine viability of cells in culture the CellTiter-Glo^® ^Luminescent Cell Viability Assay (Promega) was used. Cells were seeded in triplicate in 48-well tissue culture plates and stimulated for 48 h with DA (10 μM). The kit reagents were added directly to the cells in the well plate and luminescence of luciferase reaction, as a marker of cell viability, was measured with FLUOstar OPTIMA (BMG LABTECH GmbH, Offenburg/Germany).

### Rat tissues

Ovaries from four adult, cycling Sprague-Dawley rats (Charles River, Sulzfeld Germany) [26] were quickly removed and processed as described previously [26]. The phases of the estrus cycles were not recorded. All methods unless specified, were previously described in detail [3,9,12,13,27,28].

### In situ hybridization (ISH)

The ISH procedures were performed as described previously [26,28,29]. Briefly, cryostat sections of ovaries were probed with a digoxigenin-uridine triphosphate labeled cRNA antisense or for control purposes, with sense probes. Templates for generation of riboprobes were partial RT-PCR derived cDNAs: rat D_2 _(291 bp partial cDNA subcloned in pGEM3Z; corresponding to position 1602–1893; see [30], Genbank accession NM_012547) and rat D_1 _(396 bp in pSP72, corresponding to 484–880; see [31], Genbank accession M35077). These templates were a gift from S. Ojeda to A. Mayerhofer. Riboprobes were transcribed with T7 RNA polymerase (Promega) or with SP6 RNA polymerase (Promega). Experiments were performed using sections of 4 individual rat ovaries.

### Statistics

Data are expressed as mean + SEM unless indicated otherwise in the text and graphs. Statistical significance of changes was determined using ANOVA and Newman-Keuls's post test (Ca^2+ ^measurements) and by paired Student's t-test (comparison of cell volumes and ATP assay), performed with GraphPad Prism version 4.0a for Macintosh (GraphPad Software, San Diego California USA). Statistical significance was accepted for p < 0.05.

## Results

### 1. Evidence for D_1,2,4,5 _expression in human GCs

**Figure 1:**
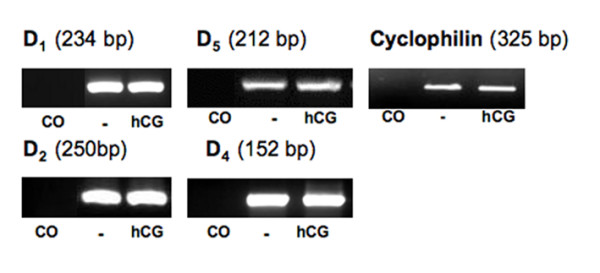
**Gene expression of DA receptors D_1,2,4,5 _in human GCs**. Ethidium bromide-stained agarose gel showing RT-PCR products, which correspond to expected size of D_1,2,4,5 _cDNAs in GCs (day 3 and day 4 of culture, with and without hCG stimulation). After sequencing they proved to be identical to the human D_1,2,4,5_. Results of PCR amplification were not visibly different between cells treated with or without hCG, as judged from similar bands and similar levels of cyclophilin, which was also amplified. Control (CO) was without input mRNA in RT-PCR reactions. The gel shows results obtained from one of four experiments that yielded comparable results.

**Figure 2:**
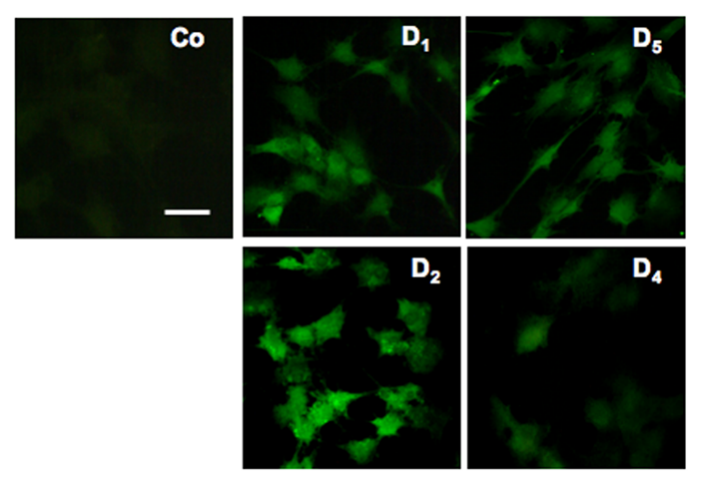
**D_1,2,4,5 _protein detection in human GCs**. Immunofluorescence localization of the D_1,2,4,5 _proteins in cultured GCs (day 3 after isolation). Control (Co), omission of antisera. Bar is equivalent to 40 μm; antisera dilutions 1:500.

**Figure 3:**
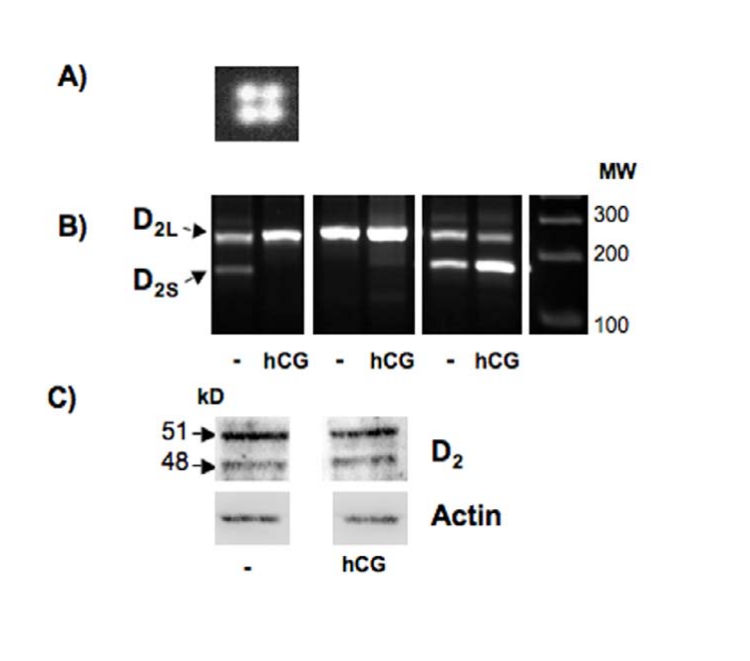
**D_2 _in human GCs**. A) Result of cDNA array, showing evidence for expression of D_2_. B) Ethidium bromide-stained agarose gel showing RT-PCR products which identify D_2L _(261 bp) and D2S (173 bp). Note that D_2L _is consistently expressed, while D_2S _expression is not depending on age or treatment with hCG (day 4 of culture, n = 4/each). C) Western blot analysis of D_2 _in cultured human GCs. Cells were treated without or with hCG for 4 days and 15 μg of total protein was analyzed. Note that the treatment has no effect on the overall levels of D_2 _protein, which can be readily detected as a double band of about 48 and 51 kDa, representing D_2S _and D_2L_, respectively. Subsequent detection of beta actin in the same blots was also performed to normalize the density of the bands. Data shown are from one of three independent experiments yielding similar results.

We tested which DA receptors are present in human GCs, i.e. a cell type derived from large preovulatory follicles, which are in the process of differentiation into luteal cells (Fig. 1, 2, 3).

RT-PCR experiments performed with mRNA obtained from GCs on the 3^rd ^or 4^th ^day of culture yielded cDNAs of expected sizes (Fig. 1), which upon sequencing proved to be identical to the known human D_1,2,4,5 _sequences. Experiments using specific primers for D_3 _did not yield results. The presence of hCG in the medium did not alter levels of DA receptor D_1,2,4,5 _mRNAs, as judged from semiquantitative RT-PCR experiments when cyclophilin was used for normalization (Fig. 1).

Results of gene array experiments provided evidence for the presence of D_1,2,5 _(D_4 _was not represented on the array membrane; D_3 _was found to be not expressed; data not shown except for D_2_, see Fig. 3A).

By immunocytochemistry D_1,2,4,5 _receptors proteins were also demonstrated in virtually all GCs using specific D_1,2,4,5 _receptor antisera. D_1,2,4,5 _immunoreactivities were associated mainly with the cytoplasm of all GCs, and all controls performed were negative (Fig. 2). D_2 _immunoreactivity appeared to be strongest.

#### D_2 _receptor splice variant

For the human D_2 _we found evidence of the long splice variant, D_2L_, and also the short form was obtained using semi-nested second PCR steps, albeit the presence of this splice variant was inconsistent and appeared not to be associated with treatment or time in culture (Fig. 3B). Both splice variants were found also at the protein level by Western blot (Fig. 3C).

### 2. Evidence for D_1,2 _receptor gene expression in rat ovary

**Figure 4:**
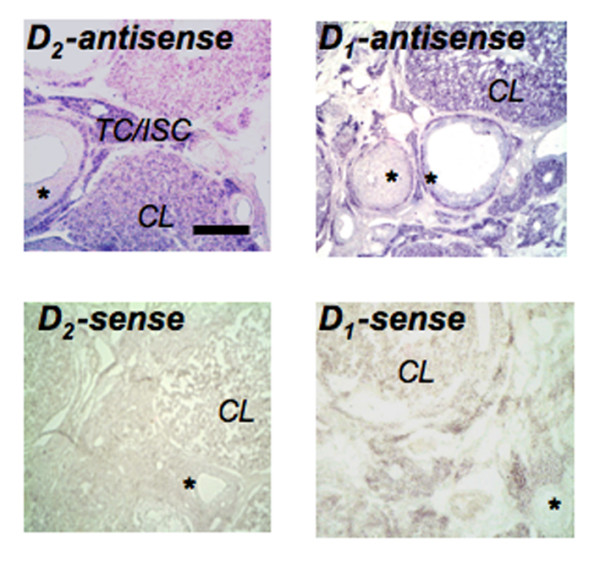
***In situ *hybridization – rat ovary**. Non-radioactive *in situ *hybridization experiments of rat ovarian slices with D_1 _or D_2 _antisense cRNA show strongest signals in theca and interstitial cells (TC/ISC) and the corpus luteum (CL), while follicular GCs (asterisk) reveal only a moderate signal. All signals are absent from a D_1 _and D_2 _receptor sense control. Bar: approximately 100 μm.

*In situ *hybridization revealed that D_1 _and D_2 _mRNAs are present in rat ovarian endocrine cells. Signals were detectable only when antisense cRNA was used, but not in sense cRNA controls. Signals were associated with GCs of follicles, but were stronger in theca and interstitial cells and the corpus luteum (Fig. 4).

### 3. Elucidation of D_2 _receptor functionality in human GCs

#### Changes of intracellular Ca^2+ ^levels in human GCs

**Figure 5:**
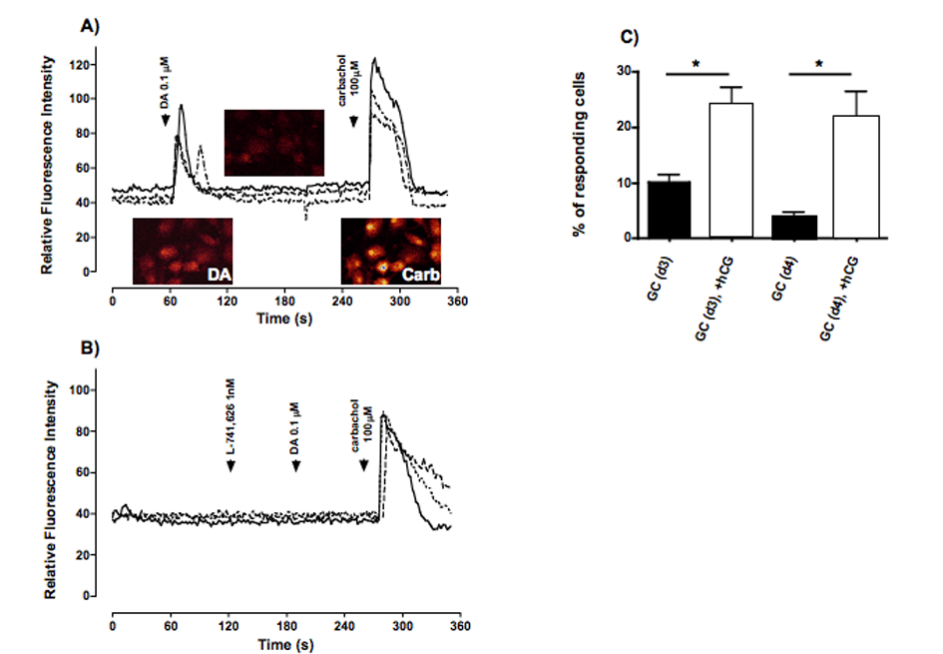
**Measurements of intracellular Ca^2+ ^concentrations in human GCs**. A) Line plot graphic showing the time-course of intracellular Ca^2+ ^changes for three representative cells sequentially treated with buffer (at t = 0), 0.1 μM DA, and 100 μM carbachol. Color images of fluo-4 AM loaded GCs of identical viewing areas are also provided. Color changes from red/yellow to yellow/blue represent increased intracellular Ca^2+ ^levels. B) Line plot graphic showing the time-course of intracellular Ca^2+ ^changes for three representative cells sequentially treated with buffer (t = 0), 1 nM L-741,626, 0.1 μM DA, and 100 μM carbachol, as a positive control. Data are expressed as relative fluorescence intensities corresponding to a pseudo color scale from red/yellow to yellow/blue. Experiments were repeated seven times with a total of 60 to 90 cells each examined in detail. C) Percentage of GCs responding to DA with increased intracellular Ca^2+ ^levels. The graphic represents the percentage of GCs (day 3 or 4 of culture) responding to DA (0.1 μM) with an increase in the intracellular Ca^2+ ^levels. GCs were either untreated or stimulated with hCG (10 IU/ml) since the 1^st ^day of culture. Experiments were repeated seven times with a total of 60 to 90 cells each examined in detail. Data represent means + SEM (ANOVA/post-test *p < 0.05).

The functionality of D_2 _(both S and L splice forms) is linked to intracellular Ca^2+ ^levels, which we thus monitored in human GCs. The addition of DA (0.1 μM) rapidly increased intracellular Ca^2+ ^levels (Fig. 5A). The number of the cells responsive to DA was lower than in the positive control, used for methodological comparison, namely the acetylcholine analogue carbachol [21].

Furthermore, the specificity of the DA action was shown by using the D_2 _blocker L-741,626 (1 nM), which prevented the Ca^2^+ increases (Fig. 5B; n = 7 experiments with 60–90 cells each). The response was not depending on the presence of extracellular Ca^2+ ^in the medium, indicating release from intracellular sites (data not shown, n = 4 experiments with 20–50 cells each).

Interestingly, the numbers of cells responsive to DA by intracellular Ca^2+ ^elevations were significantly (p < 0.05) higher when GCs were cultured in the presence of hCG, as compared to untreated cultures. The percentage of responding GCs to DA without hCG stimulation was 10.2% and 3.7% on the 3^rd ^and on the 4^th ^day of culture, respectively. After hCG exposure the percent of reacting cells was 24% and 21% on day 3 and 4, respectively, indicating a role of hCG in promoting D_2 _functionality (Fig. 5C).

### 4. Effects of DA on cell viability and volume of human GCs

**Figure 6:**
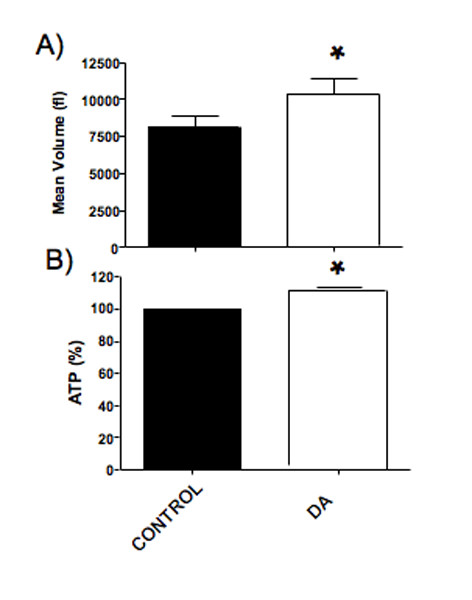
**Effect of DA on mean cellular volume and viability**. A) DA (10 μM) significantly (p < 0.05) increased the cellular volumes of GCs (means + SEM of 4 experiments; 48 h). B) The treatment of human GCs with DA (10 μM; 48 h) resulted in significantly increased ATP levels (p < 0.05; means + SEM of 3 experiments).

Human GCs exposed to DA (10 μM) for 48 h significantly increased the cell volume (Fig. 6A; control: 8085 ± 906 femto liters (fl); DA 10 μM: 10389 ± 1053 fl; n = 4, p < 0.05; determined with CASY-TT). Furthermore, the luciferase ATP assay showed a significant increased signal when the GCs were stimulated for 48 h with DA (Fig. 6B; control: 100%; DA 10 μM: 108.7 ± 0.7 %; n = 3, p < 0.05). In this assay the signal is proportional to the amount of ATP present, which correlates with the number of viable cells and/or their size. Concomitant increases in cellular volume and in ATP levels imply that the average sizes of viable cells is increased after treatment with DA. Co-expression of multiple DA receptors on each cell and lack of specific tools to distinguish between the different DA receptor members in primary cells, kept us from further investigations.

## Discussion

Catecholamines, especially NE, have long been implicated in the control of ovarian function [1-9], but whether the catecholamine DA has a specific function within the ovary is not known. A prerequisite for such an action is the presence of functional receptors in the ovary. Our results show that most of the DA receptors are present in human GCs and that two members of each DA receptor family linked to different signaling pathways are co-expressed. Our results also show expression of two prototype DA receptor family members (D_1_/D_2_) in rat ovarian endocrine cells. This resembles the previously shown expression in horse ovary and extend our studies in human and monkey ovary [14].

For our studies we employed cultured human GCs, which stem from the preovulatory follicle and are undergoing luteinization. These like monkey granulosa and luteal cells, express D_1 _[12,13] and as we found also D_5_. A previous study has shown that this receptor is linked to cAMP and DARPP-32 phosphorylation [12,13]. D_1 _and D_5 _together are known also as the D_1_-like subfamily of DA receptors, which is linked to Gs and to a similar signaling pathway. It is possible that due to co-expression of D_1_/D_5_, which we have now observed, parts of the effects ascribed previously to D_1 _are actually due to (co)-activation of both receptors. The agonists/antagonist available in the concentrations we can use in our model, does not allow to distinguish between these subtypes or draw further conclusions [32-34].

The other D_2_-like subfamily of DA receptors consists of D_2,3,4_, of which two were found in human GCs, as well. D_2 _appeared to be strongly expressed, as judged from our results using a gene array, as well as immunocytochemistry and Western blotting. This subtype is also expressed in horse ovarian endocrine cells [14] and in rat follicles and corpus luteum (this study). We therefore chose to investigate this receptor subtype further.

That D_2 _mRNA can be alternatively spliced became evident in our study, as well. Evidence for constant expression of the long form splice variant (D_2L_) was obtained, while the short form (D_2S_) was found in an inconsistent manner and appeared not to be associated with time in culture. The antiserum used for both immunocytochemistry and Western blot detects both forms and either experiment indicated ubiquitous presence and high levels in all cultured GCs. Effects of hCG on mRNA and protein levels of this subtype or on possibly splice variants were also not obvious with the experimental approach chosen, and no changes were observed with regard to the expression of the other DA receptors in GCs, as well.

It is well established that action of DA on either splice variant of the D_2 _is linked to a phosphatidylinositol-linked mobilization of intracellular Ca^2+ ^[35]. Although all cells appeared to express the receptor protein, we found when we measured the Ca^2+ ^response of individual cells to DA that only a percentage of the cultured cells showed indeed the expected changes. Furthermore, although we did not detect changes in overall levels of D_2 _in the presence of hCG in the culture medium, the percentage of responding GCs was significantly higher under these circumstances as compared to untreated cultures. The dependency on culture time or the presence of hCG indicate a relationship of the cellular differentiation state to D_2 _function and a role of the trophic hormone hCG/LH in promoting D_2 _functionality. Detailed insights e.g. at what level this may occur, are currently not possible and it remains, likewise, to be investigated whether possible transactivation of the D_2_, e.g. by EGF receptor [36] or other as yet unknown interactions may be involved.

In human the two splice variants, D_2S _and D_2L_, exhibit differences in the length of the third cytoplasmic loop involved in the coupling to G proteins [23,24] and intracellular signaling [37,38] other than phosphatidylinositol-linked mobilization of intracellular Ca^2+^. For example in neurons, D_2L _which is constantly present in human GCs, is in general also associated with inhibition of cAMP synthesis, stimulation of the MAP kinase pathway, activation of G protein coupled inwardly rectifying potassium channels (GIRKs), inhibition of Ca^2+ ^channels, potentiation of arachidonic acid release and increased Na^+^/K^+^-ATPase activity [39,40]. In the present report on this subject we were not able to address the full plethora of possible actions, but focused on effects of DA to mobilize Ca^2+^, which, by use of a specific D_2 _antagonist, were clearly shown. The Ca^2+ ^mobilizing effect of DA was not dependent on the presence of extracellular Ca^2+^, i.e. it did most likely not involve opening of Ca^2+ ^channels in the plasma membrane.

To summarize, human GCs possess four of the five known DA receptors, one of which exists in two possible splice variants and co-expression of these receptors is likely. Possible interactions of these DA receptors for example at the intracellular level (including DARPP-32) are to be assumed. Unfortunately the pharmacological tools available for the study of DA receptors in primary human GCs are limited [32-34], and antagonists/agonists interact often in a concentration-dependent manner with several receptors. Other approaches including siRNA are not readily possible for these primary cells. Clearly, the study of such interactions would be of great interest, for example with regard to DARPP-32, previously detected in human GCs and ovarian endocrine cells [12,13,18] or recently also in other endocrine tissue [41].

Therefore, a complete answer to the naive question about a specific role of DA in the ovary, which we posed, can not be given and may turn out to be much more complex than anticipated. Yet, a partial answer is provided by the results of the present study. By showing that four of the five known DA receptors are present in endocrine ovarian cells, they clearly indicate that DA is more than a precursor of NE (see Introduction). Ovarian endocrine cells are thus newly identified targets of ovarian DA and we assume based on our current results complex interactions between different DA receptor signaling cascades. Our study only provides first hints to an outcome of such actions, which implies a "trophic" role of DA for these cells. We found that treatment of cultured cells for up to 48 h with DA increased their size and viability, suggesting that DA may serve as a "differentiation factor". In brain, trophic actions of neurotransmitter are established [42,43]. Insights into actions of yet another prototype neurotransmitter present in ovary, namely acetylcholine, which stimulates GCs proliferation and differentiation, supports the notions that neurotransmitters may act as local growth factors outside the brain [44,45].

A role for ovarian DA is likely not restricted to GCs or endocrine cells, as other ovarian cells (e.g. oocytes, [46] or others) may have DA receptors or DA transporters, as well. It remains also to be investigated, whether ovarian microvasculature cells as reported in other tissues are affected by DA, which could be a profound modulator of VEGF actions and angiogenesis [46].

The presence of high ovarian DA levels on one side [1,47] and of functional DA receptors on ovarian endocrine cells linked to different second messenger pathways, on the other side, imply as yet unknown ovarian signaling systems and specific roles of DA also in the human ovary. Furthermore, existence of the DA transporter and DBH in oocytes [3] or luteal cells [3], indicate actions of DA as a precursor for NE, within the ovary.

Taken together, these results may be of as yet unforeseeable relevance to human ovarian function. This assumption is based for example on the interaction of many drugs [37] with components of the DA and the NE system, including haloperidol, cocaine and others, which may be able to affect the ovary directly. It also remains to be studied, whether like in brain, DA and steroid hormone signaling may converge, a possibility shown for DA and progesterone [48]. Finally, at least in animal studies, catecholamines (mainly NE) have been implicated in polycystic ovarian syndrome (PCOS; see [47]). Whether NE and specifically the catecholamine DA and ovarian DA receptors are involved in human PCOS, a common cause of infertility in women, remains to be studied. Our pilot studies indicate that in GCs from PCOS patients DA receptors are expressed (unpublished).

## Conclusion

The present study shows that DA receptors are expressed by rat ovarian endocrine cells. Human GCs possess four of the five known DA receptors, one of which exists in two possible splice variants, and co-expression of these receptors is likely. Our results provide a basis for future studies addressing the role of these receptors for the ovary in health and disease.

## Competing interests

The author(s) declare that they have no competing interests.

## Authors' contributions

VR-A performed most of the cellular and molecular experiments, contributed to the analysis and interpretation of data and to the drafting of the manuscript. NL performed in situ hybridization experiments and was involved in writing of the paper. DB and UB provided human GCs, were involved in the study design and contributed to the writing of the manuscript. LK was involved in conception of the study, Ca^2+ ^measurements, statistical analysis of data and writing of the paper. AM conceived of the study, coordinated most of the experiments, was involved in analysis and interpretation of the data and the writing of the paper. All authors read and approved the final manuscript.
